# Silibinin Alleviates Cognitive Dysfunction, Biochemical Alterations, and Hippocampal Structural Changes Induced by 5 days of Sleep Deprivation with or Without a Subsequent 24-H Recovery in Rats

**DOI:** 10.1007/s12035-026-06195-y

**Published:** 2026-10-03

**Authors:** Dalia Fathy El Agamy, Eman Shehata El-Roghy, Esam Omar Hendia, Omnia. Mohamed Abo Said, Ahmed Mohamed Gaafer

**Affiliations:** 1 Medical Physiology Department, Faculty of Medicine, Menoufia University, Menoufia, 32511 Egypt; 2 Medical Physiology Department, Menoufia National University, Menoufia, Egypt; 3 Department of Anatomy, College of Medicine, Jouf University, 72388 Skaka, Saudi Arabia; 4 Department of Histology and Cell Biology, Faculty of Medicine, Menoufia University, Shebin El Kom, 32511 Egypt

**Keywords:** Sleep deprivation, Stress hormones, Cognitive function, Silibinin, Sleep recovery, Inflammation and oxidative stress

## Abstract

Sleep deprivation (SD) impairs cognitive function through oxidative stress, neuroinflammation, and neuroendocrine dysregulation, whereas recovery sleep may not fully reverse these alterations. Silibinin possesses antioxidant, anti-inflammatory, and neuroprotective properties; however, its potential to facilitate recovery following prolonged SD remains unclear. This study investigated the effects of silibinin on cognitive dysfunction and hippocampal alterations induced by 5 days of SD, with or without a subsequent 24-h recovery period, in rats. Forty-eight adult male rats were allocated to control (*n* = 16), SD (*n* = 8), SD + recovery (*n* = 8), SD + silibinin (*n* = 8), and SD + silibinin + recovery (*n* = 8) groups. Cognitive performance was assessed using the Open Field Test, Morris Water Maze, and Y-Maze. Serum oxidative stress (MDA and SOD), inflammatory (TNF-α and IL-6), and hormonal biomarkers were evaluated. Hippocampal BDNF and CaMKII levels, histopathological changes, and the immunohistochemical expression of PCNA, synaptophysin, and GFAP were also assessed. SD significantly impaired cognitive performance (*P* < 0.05), increased oxidative stress and inflammatory cytokines (*P* < 0.01), altered stress-related hormones (*P* < 0.05), decreased hippocampal BDNF and CaMKII levels (*P* < 0.001) and induced marked histopathological and immunohistochemical changes. A 24-h recovery period partially ameliorated these alterations. Silibinin treatment significantly attenuated the SD-induced behavioral, biochemical, hormonal, histopathological, and immunohistochemical alterations (*P* < 0.05), with further improvement following recovery sleep (*P *< 0.05), restoring most parameters to levels comparable to those of the control group. These findings suggest that silibinin protects against SD-induced cognitive dysfunction and hippocampal injury by attenuating oxidative stress, neuroinflammation, and neuroendocrine disturbances while enhancing recovery during subsequent recovery sleep.

## Introduction

Sleep is a fundamental physiological process that is essential for maintaining cognitive function, memory consolidation, and overall health [1]. Chronic sleep deprivation (SD), an increasingly prevalent consequence of modern lifestyles, has emerged as a major public health concern and is associated with neurological, metabolic, cardiovascular, endocrine, and immune dysfunction [2]. Experimental and clinical studies have demonstrated that even modest reductions in sleep duration over several consecutive days induce cumulative physiological stress and cognitive deficits comparable to those observed following prolonged total SD [3]. Although these impairments have traditionally been considered reversible after short periods of recovery sleep (e.g., over the weekend), accumulating evidence indicates that recovery may be incomplete, with persistent neurobehavioral deficits and an underestimation of cognitive impairment despite sleep restoration [4]. Furthermore, experimental sleep restriction of only 2–3 h per night for several consecutive days has been shown to impair cardiovascular, endocrine, and immune functions. These findings underscore the cumulative impact of insufficient sleep and highlight the need for therapeutic strategies that enhance neurobiological and functional recovery following chronic SD [5].

Chronic SD disrupts cellular homeostasis and promotes the accumulation of allostatic load, resulting from sleep debt accrued over multiple nights of sleep restriction [3]. These effects are mediated by multiple interconnected mechanisms, including excessive oxidative stress, neuroinflammation, and dysregulation of the hypothalamic–pituitary–adrenal (HPA) axis. The increased production of reactive oxygen species (ROS), together with impaired antioxidant defenses promotes lipid peroxidation, reflected by increased malondialdehyde (MDA) levels and depletion of endogenous antioxidant defenses, including reduced superoxide dismutase (SOD) activity, while activation of inflammatory signaling pathways enhances the release of proinflammatory cytokines, including tumor necrosis factor-α (TNF-α) and interleukin-6 (IL-6) [6, 7]. These molecular alterations contribute to neuronal damage, hippocampal dysfunction, impaired synaptic plasticity, and ultimately result in learning and memory deficits, making them important therapeutic targets in chronic SD. In addition, chronic SD disrupts neuroendocrine homeostasis, resulting in alterations in adrenocorticotropic hormone (ACTH), corticosterone, adrenaline, insulin, and growth hormone, which collectively reflect activation of the stress response and metabolic dysregulation.

The hippocampus is particularly vulnerable to the detrimental effects of chronic SD owing to its critical role in learning, memory consolidation, and synaptic plasticity. Persistent sleep loss disrupts hippocampal structure and function by promoting oxidative stress and neuroinflammation, leading to neuronal injury, glial activation, impaired neurogenesis, and synaptic remodeling [7]. These pathological changes are accompanied by deficits in spatial learning and memory and are associated with reduced expression of neuroplasticity-related molecules, including brain-derived neurotrophic factor (BDNF) and Ca^2^⁺/calmodulin-dependent protein kinase II (CaMKII), both of which are critical regulators of neuronal survival, synaptic plasticity, and long-term potentiation [8].

Silibinin, the principal bioactive flavonolignan of silymarin extracted from the seeds of Silybum marianum (milk thistle), possesses potent antioxidant, anti-inflammatory, anti-apoptotic, and neuroprotective properties [9]. Owing to its lipophilic nature, silibinin readily crosses the blood–brain barrier and modulates multiple signaling pathways involved in oxidative stress, neuroinflammation, and neuronal survival, thereby preserving hippocampal integrity and cognitive function in various experimental models of neurological disorders [10–13]. Despite its relatively low oral bioavailability, silibinin has consistently demonstrated neuroprotective efficacy in numerous preclinical models and has exhibited a favorable safety profile in both experimental and clinical studies, supporting its therapeutic potential for neurological disorders [14]. Nevertheless, the pharmacokinetic basis underlying its central actions remains incompletely understood. However, its potential to mitigate chronic SD-induced hippocampal dysfunction and enhance neurobiological recovery during subsequent recovery sleep has not been fully elucidated.

To the best of our knowledge, although the neuroprotective effects of silibinin and the detrimental consequences of chronic SD have been investigated separately, its potential to enhance neurobiological recovery during the early recovery period following chronic SD remains largely unexplored. Therefore, the present work aimed to investigate whether silibinin could attenuate chronic SD-induced cognitive impairment and the associated oxidative, inflammatory, neuroendocrine, histopathological, and molecular alterations, as well as promote recovery from these changes during a subsequent 24-h recovery period in rats.

## Material and Methods

### Experimental Animals

Forty-eight adult male Wistar albino rats of a local strain, weighing 160–180 g, were used in this study. The rats were housed in stainless-steel cages under controlled environmental conditions (25 ± 2 °C) with a 12-h light/12-h dark cycle and free access to standard laboratory chow and water (ad libitum). Rats were acclimatized to the animal facility for 1 week before the start of the experiment. Throughout the study, the animals were maintained in the Animal Unit of the Faculty of Medicine, Menoufia University. All experimental procedures were conducted in accordance with the Guide for the Care and Use of Laboratory Animals and were approved by the Research Ethics Committee, Faculty of Medicine, Menoufia University (Approval No. = 6/2022 PHYS 26).

### Experimental Design

Rats were divided into the following experimental groups:

1. Control groups (*n* = 16 rats): rats were maintained on a large platform (15 cm in diameter) for 20 h/day, and received 1 ml of 0.5% carboxymethyl cellulose (CMC) in distilled water orally every morning for 5 days. To provide time-matched controls for the experimental groups, the control animals were divided into two subgroups (*n* = 8 each). The first subgroup was assessed on day 5 and served as the time-matched control for the SD and SD + Sil groups, whereas the second subgroup remained in their home cages for an additional 24-h and was assessed on day 6, serving as the time-matched control for the SD + Recovery and SD + Sil + Recovery groups.
2. Sleep-deprived untreated group (SD) (*n* = 8): each rat in this group was maintained on a small, raised platform (with a diameter of 6.5 cm) for 20 h/day with 4 h (7:00 am–11:00 am) rest each day for 5 days. The timing of the SD protocol was selected to ensure standardized implementation and continuous monitoring under the operational conditions of the animal facility, and was based on previously described protocols [15–19]. The same schedule was applied consistently across all experimental groups to ensure experimental uniformity.
3. Sleep-deprived untreated group with recovery (SD + R) (*n* = 8): each rat of this group were maintained on a small, raised platform (with a diameter of 6.5 cm) for 20 h/day with 4 h (7:00 am–11:00 am) rest each day for 5 days and they were allowed 24 h of undisturbed recovery sleep prior to endpoint measurements. A 24-h post-deprivation recovery period was selected because recovery sleep within this timeframe induces significant homeostatic, neurophysiological, and behavioral responses in rats. However, recovery remains incomplete even after prolonged sleep, highlighting the relevance of assessing early recovery [17, 20–22].
4. Sleep-deprived group treated with silibinin (SD + Sil) (*n* = 8): Rats were subjected to the SD protocol and received silibinin treatment [23, 24].
5. Sleep-deprived group treated with silibinin followed by recovery (SD + Sil + R) (*n* = 8): rats were subjected to the SD protocol, received silibinin treatment, and were allowed 24-h of undisturbed recovery sleep before endpoint assessments [17, 20, 23, 24].

### Silibinin Administration

Silibinin powder (Sigma-Aldrich, St. Louis, MO, USA; Cat. No. S0417) was suspended in 0.5% CMC in distilled water immediately before administration. The suspension was prepared fresh daily and mixed thoroughly before each administration. Rats in the SD + Sil and SD + Sil + R groups received the suspension orally by gastric gavage immediately after preparation at a dose of 100 mg/kg body weight once daily for five consecutive days, approximately 30 min before initiation of the SD protocol. The selected dose was based on previous studies demonstrating the neuroprotective effects of silibinin in experimental models associated with cognitive impairment, hippocampal neuronal injury, oxidative stress, and neuroinflammation [23, 24]. In particular, previous studies have reported beneficial effects of silibinin at this dose in models of neuroinflammation-induced memory impairment and hippocampal injury [25–27].

### Sleep Deprivation

Sleep deprivation was induced using the modified multiple platform (MMP) method, a well-established and widely validated experimental model for prolonged SD in rodents. This method predominantly disrupts rapid eye movement (REM) sleep by exploiting the loss of postural muscle tone during REM sleep while minimizing physical restraint and invasive manipulation. Furthermore, it permits reproducible induction of SD over several consecutive days, making it suitable for investigating the neurobehavioral and molecular consequences of chronic SD [28, 29]. Rats were placed in a custom-made glass tank (120 × 40 × 40 cm) containing 10 circular platforms. The platforms were designed to support the animals while awake but to induce awakening upon the onset of REM sleep, when loss of muscle tone caused contact with the surrounding water. Animals were able to move freely by jumping from one platform to another. Before the experimental protocol, rats were acclimatized to the apparatus for 1 h/day over three consecutive days. Following acclimatization, the glass tank was filled with water to a level approximately 3 cm below the top surface of the platforms. Food pellets and water bottles were placed above the platforms, allowing ad libitum access to food and water throughout the SD period while enabling unrestricted movement between platforms. The SD protocol was adapted from previous studies [15, 17–19]. Rats normally require approximately 12.6 h of sleep per day, with about 65–80% of total sleep occurring during the light phase and 20–35% during the dark phase [30]. Therefore, rats were subjected to SD for 20 h/day for 5 consecutive days and were allowed a 4-h sleep opportunity (7:00–11:00 a.m.) each day. This protocol was selected to induce substantial cumulative sleep loss while minimizing excessive mortality and nonspecific physiological stress associated with continuous total SD [17, 20–22].

### Behavioral Tests

#### Morris Water Maze Test

The Morris Water Maze (MWM) test was used to evaluate spatial memory retention in rats. The apparatus consisted of a circular pool (150 cm in diameter and 62.5 cm in depth) filled with opaque water maintained at 22 ± 1 °C and divided into four equal quadrants, with starting points designated as north, south, east, and west. A circular escape platform was submerged 1 cm below the water surface in the center of one quadrant, rendering it invisible to the animals. Rats underwent four consecutive days of acquisition training, during which four trials were performed daily to familiarize the animals with the platform location. The acquisition sessions were conducted solely for training purposes and were not included in the statistical analysis. In each trial, rats were released from one of the four predetermined starting positions in a fixed order, and the escape latency (time required to locate the hidden platform) was recorded. If a rat failed to find the platform within 60 s, it was gently guided to the platform and allowed to remain there briefly. Two hours after the final acquisition session, a probe trial was performed. During the probe trial, the platform was removed, and the latency to first reach the former platform location was recorded as an index of spatial memory retention and used for statistical analysis [31].

#### Y Maze Test

Spatial working memory and exploratory behavior were assessed using the Y-Maze spontaneous alternation test. The test is based on rodents’ innate preference to explore a novel arm rather than one that has been recently visited, thereby reflecting spatial working memory. The Y-Maze consisted of three identical arms labeled A, B, and C. At the start of the test, each rat was placed at the distal end of arm B, which was closest to the experimenter and near the center of the maze, and allowed to explore freely for 8 min. Arm entries were recorded when all four limbs entered an arm, and the sequence of arm visits was documented throughout the testing period. The experimenter remained at a distance from the maze, minimized noise and movement, and continuously monitored the animal’s behavior. If a rat remained immobile in a single arm for more than 60 s without exhibiting exploratory behavior, it was gently guided toward the center of the maze, and the trial was continued. The maze was cleaned and fully dried between trials to eliminate olfactory cues. Exploratory activity was quantified as the total number of arm entries during the 8-min session. Spatial working memory was evaluated by calculating the spontaneous alternation (%) as follows: (Number of spontaneous alternations ÷ [Total number of entries − 2]) × 100. Because spontaneous alternation depends on the animal's ability to remember recently visited arms, it primarily reflects hippocampal-dependent spatial working memory, with contributions from the prefrontal cortex [32].

#### Open Field Locomotion Test

Anxiety-like behavior, locomotion, and exploratory activity were assessed using the Open Field Test. The apparatus was a wooden square arena measuring 80 cm per side and 40 cm in height, with the floor divided into 16 equal squares (4 × 4) marked with white lines. Each rat was individually placed in the center of the open field and allowed to explore freely for 5 min, while its behavior was recorded using a digital video camera. Between trials, the arena was cleaned with 10% ethanol to eliminate olfactory cues. Behavioral parameters analyzed included the time spent in the central area (TCA) and number of returns to the center (NRC), the rearing (standing on hind limbs), grooming (licking body and paws, and face washing), and defecation (fecal droppings) number, which indicates the anxiety state of the animals. Exposure to a novel environment elicits an innate conflict between exploration and avoidance, making the Open Field Test a widely used paradigm for assessing anxiety-like behavior in rodents [33, 34].

All behavioral assessments were performed by the same experimenter, who was blinded to the study design and experimental groups.

### Blood Sampling

At the end of the experimental period, animals in each group were subjected to neurobehavioral testing. Subsequently, the rats were fasted overnight, and retro-orbital blood samples (2 ml) were collected by trained personnel from each animal into sterile tubes. Immediately thereafter, the animals were euthanized by cervical dislocation in accordance with the approved institutional ethical guidelines. Blood samples were allowed to clot and then centrifuged to obtain serum for biochemical analysis.

### Tissue Collection

Following euthanasia, the brains were immediately excised, bisected, and the hippocampi were processed for homogenization, histopathology and immunohistochemical analyses. Animal remains were disposed of in a biosecure and hygienic manner in accordance with institutional guidelines.

### Biochemical Analyses

Following behavioral assessments, collected plasma, serum, and hippocampal tissue samples were used for biochemical analyses. Plasma concentrations of ACTH (CUSABIO Biotech Co., Wuhan, China; Cat. No. CSB-E08967r), corticosterone (Cayman Chemical, Ann Arbor, MI, USA; Cat. No. 501320), adrenaline (Elabscience Biotechnology Inc., Wuhan, China; Cat. No. E-EL-R3019), growth hormone (Millipore, Billerica, MA, USA; Cat. No. EZRMGH-45K), and insulin (Sigma-Aldrich, Germany; Cat. No. RAB0904) were determined using commercially available ELISA kits. Serum concentrations of TNF-α (R&D Systems, Minneapolis, MN, USA; Cat. No. SRTA00) and IL-6 (R&D Systems, Minneapolis, MN, USA; Cat. No. R6000B) were quantified using Quantikine® ELISA kits, and absorbance was measured using an automated microplate reader (SUNRISE Touchscreen, TECAN, Salzburg, Austria). Oxidative stress parameters, including MDA (Cat. No. MD2529) and SOD (Cat. No. GR2511), were assessed using enzymatic colorimetric assay kits supplied by Biodiagnostic (Giza, Egypt). Furthermore, hippocampal tissue homogenates were prepared to determine BDNF (Boster Biological Technology, Pleasanton, CA, USA; Cat. No. EK0308) and CaMKII (BlueGene Biotech, Shanghai, China; Cat. No. E02C0682) using commercially available ELISA kits supplied by Bio-KITS (Cairo, Egypt), according to the manufacturers' instructions.

### Histopathological Assessment

One hemisphere of the brain was harvested from each rat for histological examination. Brain tissues were fixed in 10% formol saline and processed to prepare 5-µm-thick paraffin sections. Serial sections at the level of the hippocampus were prepared, and approximately 10 sections per animal were examined. They were used for H&E staining for monotonous histological examinations [35].

### Immunohistochemical Study

The immunohistochemical expression of the following markers was assessed: glial fibrillary acidic protein (GFAP) as a marker of astrogliosis [36, 37], proliferating cell nuclear antigen (PCNA) as a marker of cell proliferation or DNA repair [38], and synaptophysin as a marker of synaptic plasticity [39].

Immunohistochemical staining was performed on 4-mm thick sections using a streptavidin–biotin complex immunoperoxidase system. Serial sections were deparaffinized on charged slides, incubated in 0.1% hydrogen peroxide for 30 min (to block endogenous peroxidase) and then incubated overnight at room temperature with the following primary antibodies: anti-GFAP (monoclonal mouse Cat. No. MS-280-R7, Lab Vision Corporation, Fremont, USA) [40], anti-synaptophysin antibody (monoclonal mouse MAB5258; Chemicon, Temecula, CA, USA) [41], anti-PCNA antibody (clone PC 10; Dako Denmark A/S,Glostrup, Denmark) [42], Finally, the slides were washed with diluted phosphate-buffered saline (PBS) and incubated with secondary anti-mouse antibody universal kits for approximately 30 min at room temperature. The control slides were included in each run. Negative controls were prepared by exclusion of the primary antibody.

### Morphometric Study

All measurements were carried out on 10 non-overlapping sections for each group at the same magnification. The study was performed using a Leica Qwin 500 LTD software image analysis computer system (Cambridge, UK). The subsequent parameters were measured: (1) the mean thickness of the pyramidal layer for CA1 (um) was measured in H&E-stained sections (× 200); (2) the area percentage of GFAP immunostaining per field (× 200); (3) the average number of positive PCNA immunostained neurons per field (× 200); and (4) area percentage for synaptophysin immunostaining per field (× 400) [43, 44]. The calculated data were analyzed statistically to allow comparisons among groups.

### Statistical Analysis

The data were analyzed using SPSS, version 20, (SPSS, Inc., USA). Data were assessed for normality using the Shapiro–Wilk test. All data variables were normally distributed (*P > *0.05); therefore, parametric statistical analyses were performed. The findings were presented in the form of mean ± standard deviation (SD). A post hoc Tukey test was performed after a one-way analysis of variance (ANOVA) to determine the significance of the differences between the groups. After calculating the *P *values, a statistical difference was considered significant if *P* ≤ 0.05.

## Results

### Neurobehavioral Assessment

#### Morris Water Maze

**Fig. 1:**
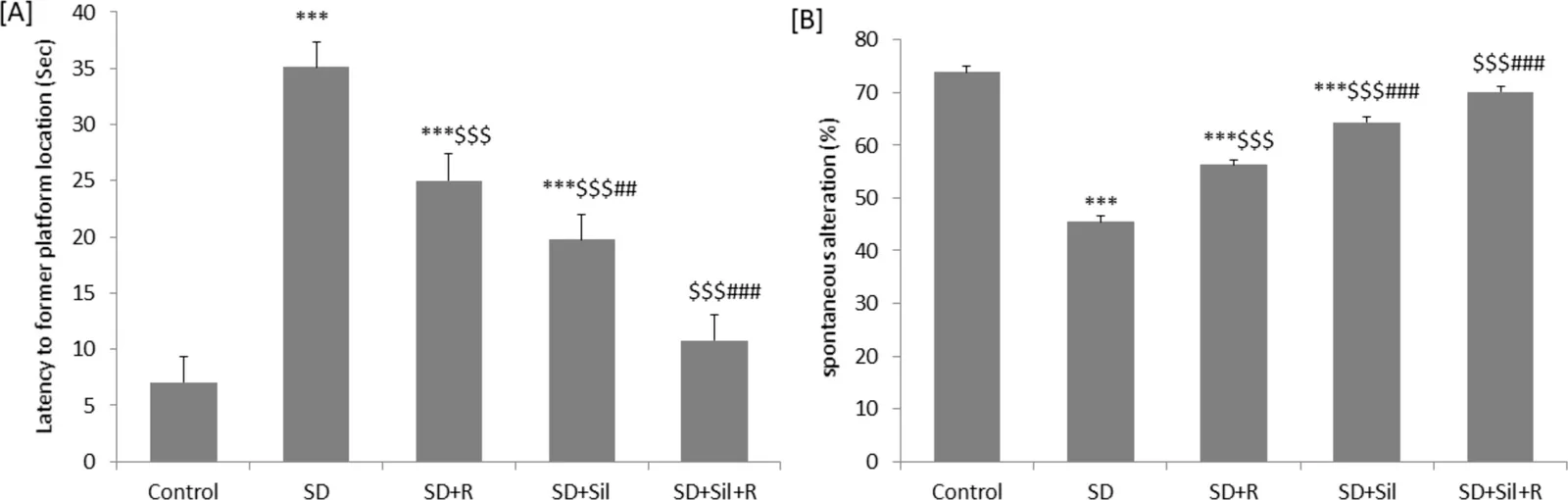
Effect of Silibinin on behavioral and cognitive performance in the different studied groups. **A** Latency to first reach the former platform location during the probe trial of the MWM test (Sec). **B** Percentage of spontaneous alternation in the Y-Maze test (%). Data are represented as mean ± SD (*n* = 8), group comparisons were performed using one-way ANOVA, followed by Tukey’s multiple-comparisons test. ***P < 0.001 versus the control group; $$$*P* < 0.001 versus the SD group; ##*P* < 0.01, and ###P < 0.001 versus the SD + R group. SD: Sleep-deprived untreated group, SD + R: Sleep-deprived untreated group with recovery, SD + Sil: Sleep-deprived group treated with silibinin, SD + Sil + R: Sleep-deprived group treated with silibinin followed by recovery

Compared with the control group, the SD group exhibited a significant increase in the latency to first reach the former platform location during the probe trial (*P* < 0.001). Both the SD + R and SD + Sil groups showed significant reductions in this latency compared with the SD group (*P* < 0.001); however, values remained significantly different from those of the control group (*P* < 0.001). The SD + Sil group also demonstrated significantly greater improvement than the SD + R group (*P* < 0.01). The greatest improvement was observed in the SD + Sil + R group, which showed a highly significant reduction in latency compared with both the SD and SD + R groups (*P* < 0.001), with values that were not significantly different from those of the control group (*P* > 0.05) (Fig. 1A).

#### Y-Maze

Compared with the control group, the SD group showed a significant reduction in the percentage of spontaneous alternation (*P* < 0.001). Both the SD + R and SD + Sil groups demonstrated significant improvements compared with the SD group (*P* < 0.001), although values remained significantly lower than those of the control group (*P* < 0.001). The SD + Sil group showed greater improvement than the SD + R group (*P* < 0.001). The SD + Sil + R group exhibited the highest percentage of spontaneous alternation, showing significant improvement compared with both the SD and SD + R groups (*P* < 0.001), with no significant difference compared with the control group (*P* > 0.05) (Fig. 1B).

#### Open Field Test

Compared with the control group, the SD group exhibited a significant increase in TCA (*P* < 0.001). Both the SD + R and SD + Sil groups showed significant improvement compared with the SD group (*P* < 0.001), although values remained significantly different from those of the control group (*P* < 0.001). The SD + Sil group demonstrated significantly greater improvement than the SD + R group (*P* < 0.05). The SD + Sil + R group exhibited a significant reduction in TCA compared with the SD and SD + R groups (*P *< 0.001), with values comparable to those of the control group (*P* > 0.05) (Table 1).

**Table 1:** Effect of silibinin on open field test performance following chronic SD in the different studied groups

Parameter	Control	SD	SD + R	SD + Sil	SD + Sil + R
TCA (seconds)	8.71 ± 2.37	35.12 ± 3.2 ***	23.75 ± 3.24 ***$$$	19.5 ± 3.5 ***$$$#	12.25 ± 2.8 $$$###
NRC	5.5 ± 1.4	1.75 ± 0.7 ***	3.63 ± 0.9 *$	3.75 ± 0.7 *$	5.38 ± 1.6 $$$#
Grooming frequency	3.62 ± 0.91	9.75 ± 1.6 ***	6.38 ± 1.6 *$	5.38 ± 0.9 $	4.9 ± 1.1 $
Rearing frequency	4.38 ± 1.1	8.63 ± 1.9 ***	6.12 ± 1.5 *$	5.87 ± 1.35 $	4.62 ± 1.5 $
Defecation frequency	2.38 ± 0.9	6 ± 1.9 ***	4.12 ± 0.9 *$	3.25 ± 0.8 $	2.9 ± 0.83 $

Data are represented as mean ± SD (*n* = 8). Group comparisons were performed using one-way ANOVA, followed by Tukey’s multiple-comparisons test. *P < 0.05, and ***P < 0.001 versus the control group; $*P* < 0.05, and $$$*P* < 0.001 versus the SD group; #*P* < 0.05, and ###*P *< 0.001 versus the SD + R group. *SD:* sleep-deprived untreated group, *SD + R:* sleep-deprived untreated group with recovery, *SD + Sil:* sleep-deprived group treated with silibinin, *SD + Sil + R:* sleep-deprived group treated with silibinin followed by recovery, *OFT:* Open Field Test, *TCA:* time spent in the central area, *NRC*:number of returns to the center

Regarding NRC, the SD group exhibited a significant decrease compared with the control group (*P* < 0.001). Both the SD + R and SD + Sil groups showed significant improvement compared with the SD group (*P* < 0.05), although their values remained significantly different from those of the control group (*P* < 0.05). No significant difference was observed between the SD + Sil and SD + R groups (*P* > 0.05). The SD + Sil + R group demonstrated a significant increase in NRC compared with the SD group (*P *< 0.001) and SD + R group (*P* < 0.05), with values comparable to those of the control group (*P* > 0.05) (Table 1).

When SD group was compared with the control group, it showed significantly higher frequencies of rearing, grooming, and defecation in the Open Field Test (*P* < 0.001). SD + R and the treatment groups showed significant improvement in these parameters when compared with SD group (*P* < 0.05). However, rearing, grooming, and defecation frequencies in the SD + R group remained significantly higher than those of the control group (*P* < 0.05), whereas the SD + Sil and SD + Sil + R groups showed no significant differences from the control group (*P* > 0.05) (Table 1).

### Biochemical Tests

#### Oxidative Stress Parameters (MDA and SOD)

**Fig. 2:**
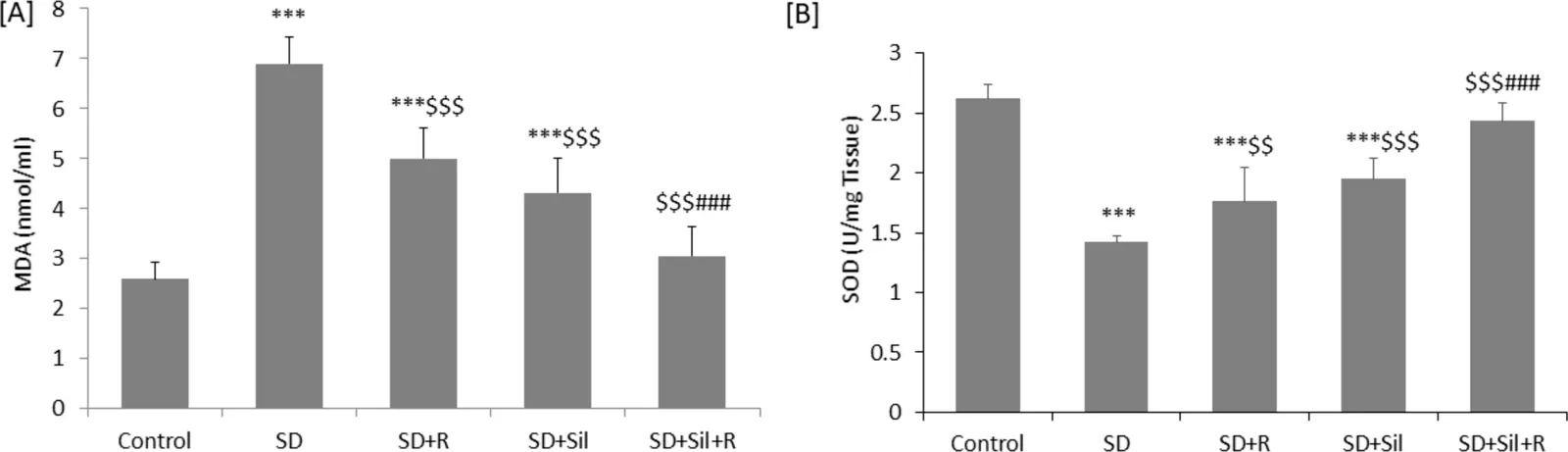
Effect of silibinin on oxidative stress markers in sleep-deprived rats with or without subsequent 24-h recovery. **A** MDA (nmol/ml) and **B** SOD (U/mg tissue). Data are represented as mean ± SD (*n* = 8), group comparisons were performed using one-way ANOVA, followed by Tukey’s multiple-comparisons test. ***P < 0.001 versus the control group; $$*P* < 0.01, and $$$*P* < 0.001 versus the SD group; ###*P *< 0.001 versus the SD + R group. SD: sleep-deprived untreated group; SD + R: sleep-deprived untreated group with recovery; SD + Sil: sleep-deprived group treated with silibinin; SD + Sil + R: sleep-deprived group treated with silibinin followed by recovery. MDA: malondialdehyde; SOD: superoxide dismutase

Serum MDA levels were significantly increased in the SD group compared with the control group (*P* < 0.001). Both the SD + R and SD + Sil groups showed significantly lower MDA levels than the SD group (*P* < 0.001), but still significantly higher than control group (*P* < 0.001). The SD + Sil group exhibited insignificant changes when compared to the SD + R group (*P* > 0.05). Furthermore, SD + Sil + R group demonstrated a significant reduction in serum MDA levels compared with the SD and SD + R groups (*P* < 0.001) with values comparable to those of the control group (*P* > 0.05) (Fig. 2A).

Serum SOD levels were significantly lower in the SD group than in the control group (*P* < 0.001). Both the SD + R and SD + Sil groups showed significantly higher SOD levels than the SD group (*P* < 0.01 and *P* < 0.001 respectively); however, their levels remained significantly lower than those of the control group (*P* < 0.001). No significant difference was observed between the SD + R and SD + Sil groups (*P* > 0.05). Furthermore, the SD + Sil + R group demonstrated a significant increase in serum SOD levels compared with the SD and SD + R groups (*P* < 0.001), with values comparable to those of the control group (*P* > 0.05) (Fig. 2B).

#### Inflammatory Markers (TNF-α and IL-6)

**Fig. 3:**
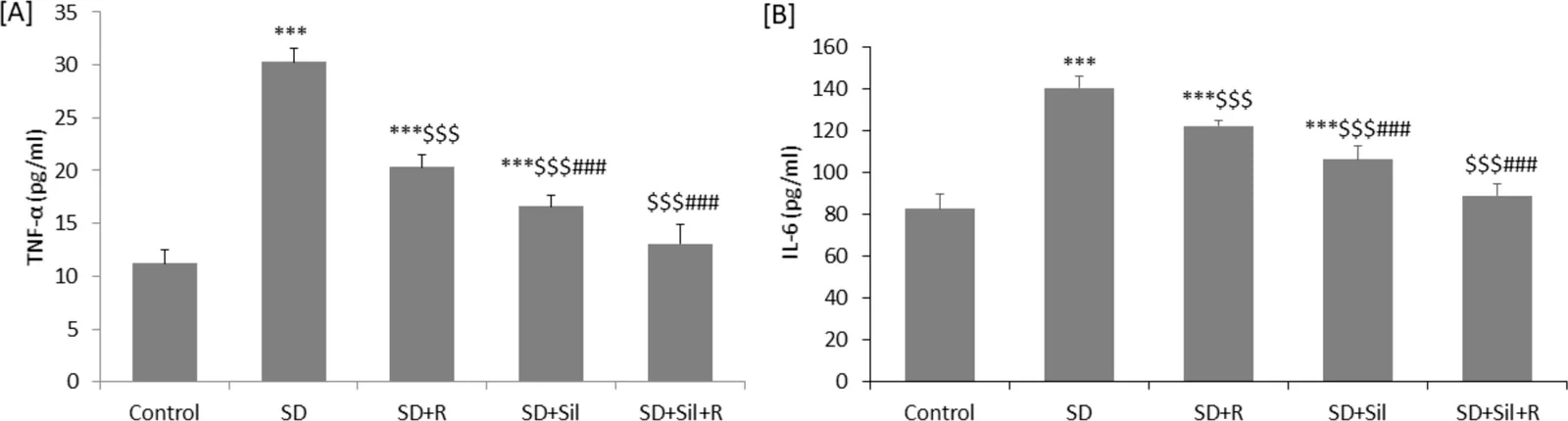
Effect of silibinin on inflammatory markers in sleep-deprived rats with or without subsequent 24-h recovery. **A** TNF-α (pg/ml) and **B** IL-6 (pg/ml). Data are represented as mean ± SD (*n* = 8), group comparisons were performed using one-way ANOVA, followed by Tukey’s multiple-comparisons test. ***P < 0.001 versus the control group; $$$*P* < 0.001 versus the SD group; ###*P* < 0.001 versus the SD + R group. SD: sleep-deprived untreated group; SD + R: sleep-deprived untreated group with recovery; SD + Sil: sleep-deprived group treated with silibinin; SD + Sil + R: sleep-deprived group treated with silibinin followed by recovery. TNF-α: tumor necrosis factor-alpha; IL-6: Interleukin-6

Sleep deprivation significantly increased serum TNF-α levels compared with the control group (*P* < 0.001). Following the 24-h recovery period (SD + R), TNF-α levels were significantly reduced compared with the SD group (*P* < 0.001); however, they remained significantly higher than those of the control group (*P* < 0.001). Treatment with silibinin (SD + Sil) further reduced TNF-α levels compared with the SD (*P* < 0.001) and SD + R groups (*P* < 0.001), although the levels remained significantly higher than those of the control group (*P* < 0.001). The SD + Sil + R group exhibited the greatest reduction in TNF-α levels, showing significantly lower values than the SD and SD + R groups (*P* < 0.001), with levels comparable to those of the control group (*P* > 0.05) (Fig. 3A).

A similar trend was observed for IL-6 levels. SD significantly increased IL-6 levels compared with the control group (*P* < 0.001). The SD + R group showed a significant reduction in IL-6 levels compared with the SD group (*P* < 0.001); however, the levels remained significantly higher than those of the control group (*P* < 0.001). Treatment with silibinin (SD + Sil) further reduced IL-6 levels when compared with the SD (*P* < 0.001) and SD + R groups (*P* < 0.001), although the levels remained significantly higher than those of the control group (*P* < 0.001). The SD + Sil + R group exhibited the greatest reduction in IL-6 levels, showing significantly lower values than the SD (*P* < 0.001) and SD + R groups (*P* < 0.001), with levels comparable to those of the control group (Fig. 3B).

### Hormonal Assays

#### ACTH, Corticosterone, and Insulin

Serum ACTH and corticosterone levels were significantly higher in the SD group than in the control group (*P* < 0.001). Both the SD + R and SD + Sil groups showed significant reductions in serum ACTH and corticosterone levels compared with the SD group (*P* < 0.001); however, their levels remained significantly higher than those of the control group in both the SD + R and SD + Sil groups (ACTH: *P* < 0.001 and *P* < 0.01, respectively; corticosterone: *P* < 0.001). The SD + Sil group showed a significant reduction in ACTH and corticosterone levels compared with the SD + R group (ACTH: *P* < 0.05; corticosterone: *P* < 0.001). Furthermore, the SD + Sil + R group demonstrated significant reductions in serum ACTH and corticosterone levels compared with the SD group (*P* < 0.001) and the SD + R group (ACTH: *P* < 0.05; corticosterone: *P* < 0.001). Both ACTH and corticosterone levels in the SD + Sil + R group were comparable to those of the control group (*P* > 0.05) (Table 2).

**Table 2:** Effect of silibinin on stress-related hormones and fasting serum insulin in sleep-deprived rats with and without subsequent 24-h recovery

	Control	SD	SD + R	SD + Sil	SD + Sil + R
ACTH (pg/ml)	150 ± 20	260 ± 30 ***	200 ± 28 ***$$$	180 ± 25 **$$$#	152 ± 18 $$$#
Corticosterone (ng/ml)	115.23 ± 8.8	277.91 ± 3.69 ***	194.63 ± 9.87 ***$$$	162.36 ± 7.50 ***$$$###	127.56 ± 8.95 $$$###
Fasting serum insulin (µIU/ml)	7.2 ± 0.4	9.3 ± 0.5 ***	8.6 ± 0.4 ***$$	8 ± 0.4 **$$$	7.7 ± 0.3 $$$#
Growth hormone (ng/ml)	10.56 ± 1.24	5.75 ± 1.46 ***	6.50 ± 1.04 ***	7.76 ± 1.13 **$	7.88 ± 1.44 *$$$#
Adrenaline (ng/ml)	0.31 ± 0.08	1.08 ± 0.26 ***	0.59 ± 0.11 *$$$	0.43 ± 0.14 *$$$	0.32 ± 0.11 $$$#

Data are represented as mean ± SD (*n* = 8). Group comparisons were performed using one-way ANOVA, followed by Tukey’s multiple-comparisons test. *P < 0.05, **P < 0.01, and ***P < 0.001 versus the control group; $*P *< 0.05, $$*P* < 0.01, and $$$*P* < 0.001 versus the SD group; #*P* < 0.05, ##*P* < 0.01, and ###*P* < 0.001 versus the SD + R group. *SD:* sleep-deprived untreated group, *SD + R:* sleep-deprived untreated group with recovery, *SD + Sil:* sleep-deprived group treated with silibinin, *SD + Sil + R:* sleep-deprived group treated with silibinin followed by recovery, *ACTH:* adrenocorticotropic hormone

Serum insulin levels were significantly higher in the SD group than in the control group (*P* < 0.001). Both the SD + R and SD + Sil groups showed significantly lower serum insulin levels than the SD group (*P* < 0.01, *P* < 0.001, respectively); however, their levels remained significantly higher than those of the control group (*P* < 0.001, *P* < 0.01, respectively). The SD + Sil group exhibited insignificant changes in insulin levels compared with the SD + R group (*P* > 0.05). Furthermore, the SD + Sil + R group demonstrated a significant reduction in serum insulin levels compared with the SD (*P* < 0.001) and SD + R groups (*P *< 0.05), with values comparable to those of the control group (*P* > 0.05) (Table 2).

#### Growth Hormone and Adrenaline

Serum growth hormone levels were significantly lower in the SD group than in the control group (*P* < 0.001). The SD + R group showed insignificant change in growth hormone levels compared with the SD group (*P* > 0.05); and the levels remained significantly lower than those of the control group (*P* < 0.001). Treatment with silibinin (SD + Sil) increased growth hormone levels compared with the SD group (*P *< 0.05). However, they remained significantly lower than those of the control group (*P* < 0.01) and did not differ significantly from the SD + R group (*P* > 0.05). The SD + Sil + R group showed the greatest improvement, with significantly higher growth hormone levels than the SD (*P* < 0.001) and SD + R groups (*P* < 0.05); however, the levels were still significantly lower than those of the control group (*P* < 0.05) (Table 2).

Serum adrenaline levels were significantly increased in the SD group compared with the control group (*P* < 0.001). Both the SD + R and SD + Sil groups showed significantly lower serum adrenaline levels than the SD group (*P* < 0.001); however, their levels remained significantly higher than those of the control group (*P* < 0.05). No significant difference was observed between the SD + R and SD + Sil groups (*P* > 0.05). Furthermore, the SD + Sil + R group demonstrated a significant reduction in serum adrenaline levels compared with the SD (*P* < 0.001) and SD + R groups (*P* < 0.05), with values comparable to those of the control group (*P *> 0.05) (Table 2).

#### Brain-Derived Neurotrophic Factor

**Fig. 4:**
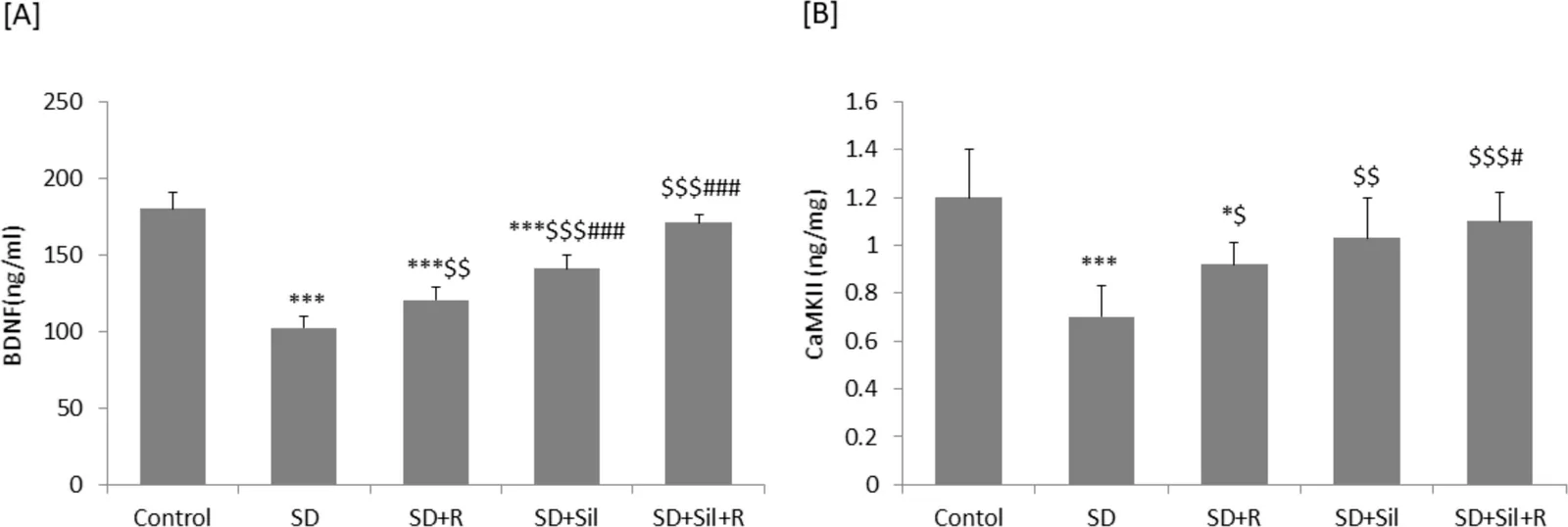
Effect of silibinin administration on **A** BDNF (ng/ml) and **B** CaMKII (ng/mg) in sleep-deprived rats with and without consequent 24-h recovery. Data are represented as mean ± SD (*n* = 8); group comparisons were performed using one-way ANOVA, followed by Tukey’s multiple-comparisons test. *P < 0.05, **P < 0.01, and ***P < 0.001 versus the control group; $*P* < 0.05, $$*P* < 0.01, and $$$*P* < 0.001 versus the SD group; #*P* < 0.05, ##*P* < 0.01, and ###*P* < 0.001 versus the SD + R group. SD: sleep-deprived untreated group; SD + R: sleep-deprived untreated group with recovery; SD + Sil: sleep-deprived group treated with silibinin; SD + Sil + R: sleep-deprived group treated with silibinin followed by recovery. BDNF: brain‑derived neurotrophic factor; CaMKII: calcium/calmodulin‑dependent protein kinase II

The SD group showed significantly lower BDNF levels than the control group (*P* < 0.001). Following 24-h recovery period (SD + R), BDNF levels were significantly increased compared with the SD group (*P* < 0.01); however, they remained significantly lower than those of the control group (*P* < 0.001). Treatment with silibinin (SD + Sil) further increased BDNF levels relative to the SD group (*P* < 0.001) and SD + R groups (*P *< 0.001), although the levels remained significantly lower than those of the control group (*P* < 0.001). Furthermore, the SD + Sil + R group exhibited the greatest increase in BDNF levels, showing significantly higher values than the SD (*P* < 0.001) and SD + R (*P* < 0.001) with levels comparable to those of the control group (*P* > 0.05) (Fig. 4A).

#### Calcium-Calmodulin-Dependent Kinase II

The SD group exhibited significantly lower CaMKII levels than the control group (*P* < 0.001). Following the 24-h recovery period (SD + R), CaMKII levels were significantly increased compared with the SD group (*P* < 0.05); however, they remained significantly lower than those of the control group (*P* < 0.05). Treatment with silibinin (SD + Sil) further increased CaMKII levels compared with the SD group (*P* < 0.01), with values comparable to those of the control group (*P *> 0.05). CaMKII levels in the SD + Sil group were not significantly different from those in the SD + R group (*P* > 0.05). Furthermore, the SD + Sil + R group exhibited significantly higher CaMKII levels than the SD (*P* < 0.001) and SD + R groups (*P* < 0.05), with values comparable to those of the control group (*P *> 0.05) (Fig. 4B).

### Histological Results

#### Hematoxylin and Eosin-Stained Sections

**Fig. 5:**
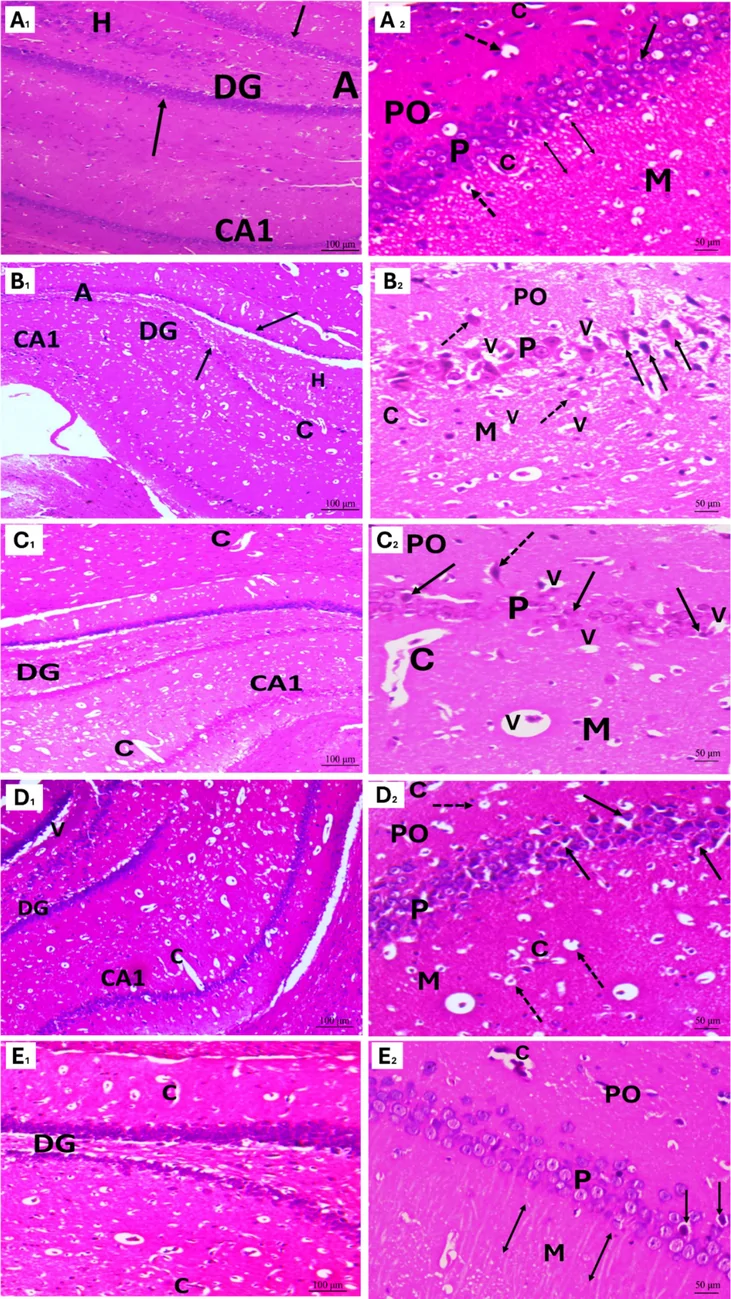
Hematoxylin and eosin (H&E)-stained sections of the hippocampus in the different studied groups (*n* = 8). **A1**–**E1**  × 100; scale bar = 100 µm. **A2**–**E2**  × 200; scale bar = 50 µm). Control group (**A1**): Well-organized DG with its two blades (arrows), apex (A) and hilum (H). The CA1 region appears normal. **A2** Zone 1 of the CA1 region, formed of outer polymorphic layer (PO), middle pyramidal layer (P), and inner molecular layer (M). The pyramidal layer consists of regular rows (3–4) of densely packed pyramidal cells with basophilic cytoplasm and large vesicular nuclei and prominent nucleoli (arrow). Their axons extend in the molecular layer, forming the neuropil (double-headed arrows). Neuroglial cells (dashed arrows) and tiny blood capillaries (C) are scattered among molecular and polymorphic layers. SD group (**B1**) DG with thin, attenuated 2 blades (arrows), apex (A), and hilum (H); CA1 of the hippocampus appears dispersed with few pyknotic cells. **B2** CA1 of the sleep-deprived (SD) group showing reduction in pyramidal layer thickness with disorganization (P). Hypocellularity with dislocation of some pyramidal cells (dashed arrows). Some pyramidal cells appear degenerated, with deeply stained pyknotic nuclei and eosinophilic cytoplasm (arrow). Some vacuolization is noticed around neurons and in the neuropil of the polymorphic layer (V). Slight capillary congestion (C) is seen. (H&E × 200). SD + R group (**C1**) CA1 of the hippocampus and the DG moderately restoring their thickness, with obvious capillary dilatation (C). **C2** Displacement of eosinophilic neuron (dashed arrow) in the polymorphic layer (PO). The pyramidal layer contains about 2 rows of misaligned pyramidal cells; some of them have small dark nuclei (arrow). Notice: congested dilated blood capillaries (C) in the molecular layer (M) and vacuolization in neurons and neuroglial cells (v). SD + Sil group (**D1**) Well-organized Zone 1 of the CA1 of the hippocampus and the DG with slight capillary dilatation (C) and vacuolization (V) (**D2**) CA1 showing an aligned pyramidal layer (P) having many neurons with dark nuclei and mildly vacuolated cytoplasm (arrow). An apparent increase in neuroglial cells (dashed arrows) and blood capillaries (C) in polymorphic (Po) and molecular layers (M) is noticed. SD + Sil + R group (**E1**) Dentate gyrus within normal structures as control group. Numerous blood capillaries (C) are noticed. **E2** CA1 resembles that of the control group, but with a few pyramidal neurons that have dark nuclei (arrows). The thickness of the pyramidal layer is restored (P). The polymorph layer (Po) contains slightly congested blood capillaries, and the molecular layer (M) contains apical dendrites of pyramidal neurons (double-headed arrow)

**Fig. 6:**
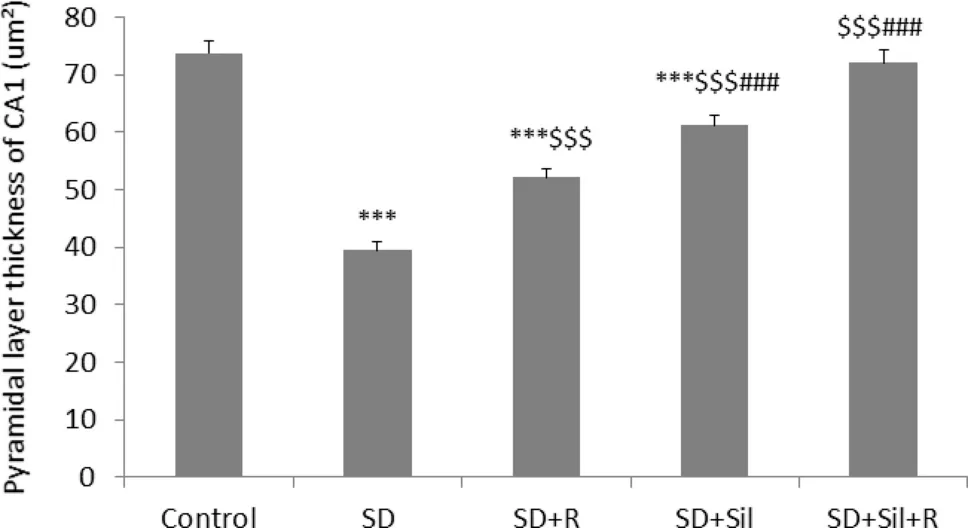
Bar chart showing the mean ± SD of pyramidal layer thickness for CA1 (µm) in all studied groups. Group comparisons were performed using one-way ANOVA, followed by Tukey’s multiple-comparisons test (*n* = 8). ***P < 0.001 versus the control group; $$$*P* < 0.001 versus the SD group; ###*P* < 0.001 versus the SD + R group. SD: sleep-deprived untreated group; SD + R: sleep-deprived untreated group with recovery; SD + Sil: Sleep-deprived group treated with silibinin; SD + Sil + R: sleep-deprived group treated with silibinin followed by recovery

Histopathological examination of hippocampal sections from the control group revealed normal histological features of the hippocampal dentate gyrus (DG) and CA1 region (Fig. 5(A1 and A2)). The SD group showed a degenerated DG with remarkable hypocellularity. This group also revealed a disorganized, thin CA1 of the hippocampus with many eosinophilic neurons and pyknotic nuclei. Vacuolization was noticed around neurons and in the neuropil of the polymorphic layer, with blood capillary congestion. There was a significant reduction in the thickness of the pyramidal layer compared to the control group (*P* < 0.001) (Fig. 5(B1 and B2); Fig. 6). Following a 24-h recovery period (SD + R), mild restoration of the thickness of CA1 of the hippocampus and the DG was observed, but some displacement of eosinophilic neurons and degenerated pyramidal cells was observed. Capillary congestion and vacuolization of neurons and neuroglial cells were noticed (Fig. 5(C1 and C2)). There was a significant increase in the thickness of the pyramidal layer compared with the SD group (*P* < 0.001), although the values remained significantly different from those of the control group (*P* < 0.001; Fig. 6). Treatment with silibinin (SD + Sil) resulted in moderate restoration of the normal organization of the CA1 region of the hippocampus and the DG. Some neurons showed pyknosis of their nuclei and slight cytoplasmic vacuolization. Neuroglial cells were apparently increased. Quantitative analysis demonstrated a significant increase in pyramidal layer thickness compared with both the SD group and the SD + R group (*P* < 0.001). However, the pyramidal layer remained significantly thinner than that of the control group (*P* < 0.001) (Fig. 5(D1 and D2); Fig. 6). The SD + Sil + R group exhibited near-normal histological architecture of the DG and CA1 region, closely resembling that of the control group, with only occasional pyramidal neurons showing darkly stained nuclei (Fig. 5(E1 and E2)). Furthermore, pyramidal layer thickness was significantly greater than that observed in both the SD group and the SD + R group (*P* < 0.001). Importantly, no significant difference was detected between the SD + Sil + R and control groups (*P* > 0.05; Fig. 6), indicating substantial restoration of CA1 pyramidal layer thickness following combined silibinin treatment and recovery.

### Immunohistochemical Results

#### GFAP Immunostaining

**Fig. 7:**
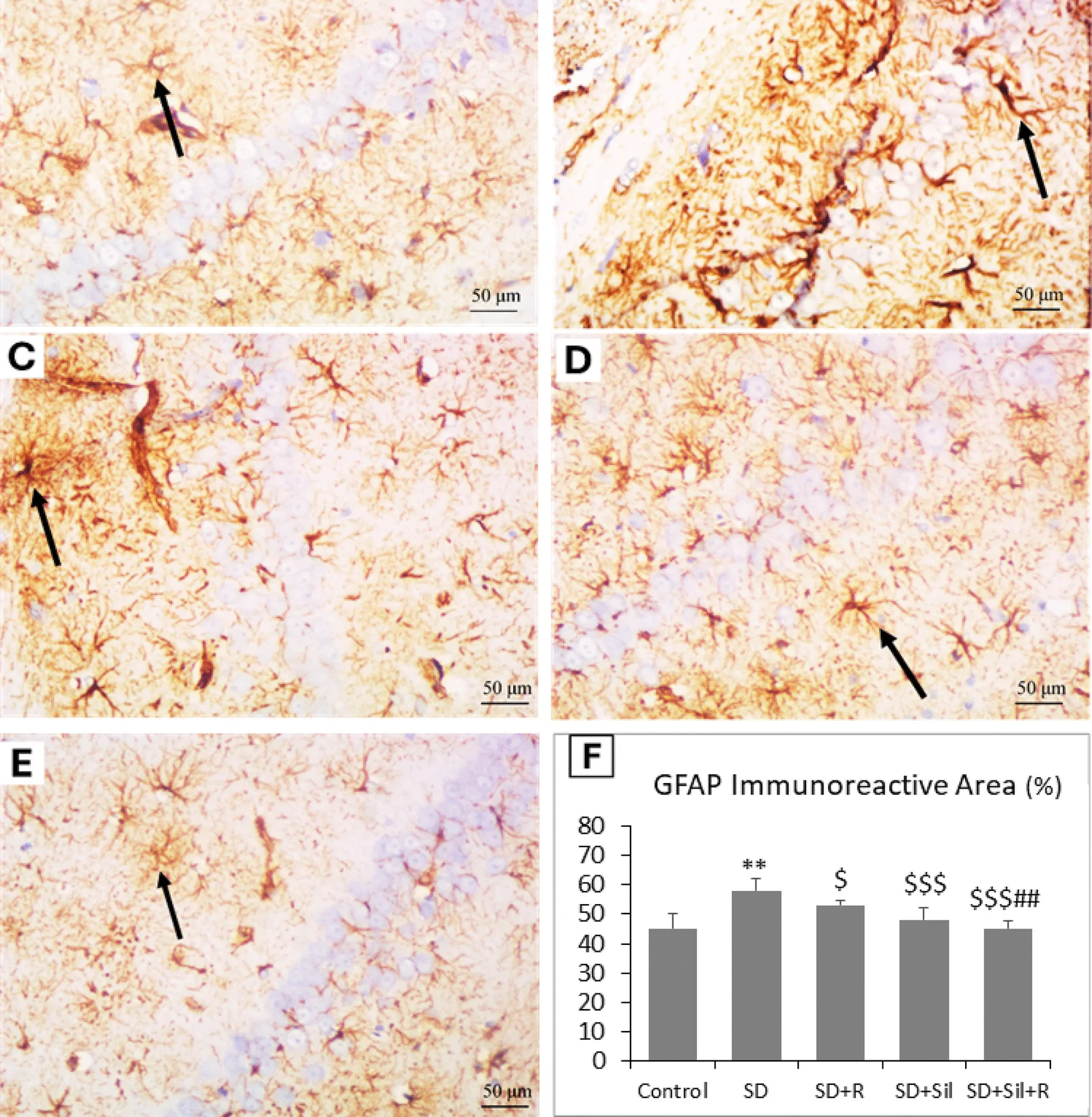
GFAP immune-stained sections the CA1 region of the hippocampus (GFAP immunostaining, × 200; scale bar = 50 µm). **A **control group showing small star-shaped astrocytes with thin processes and weak brown GFAP immunoreactivity (arrow). **B** SD group showing enlarged astrocytes with thick processes and intense brown GFAP-positive cytoplasmic immunoreactivity (arrows). **C** SD + R showing increased GFAP immunoreactivity in astrocytes (arrow). **D** SD + Sil group showing moderate GFAP cytoplasmic immunoreactivity with some enlarged astrocytes (arrows). **E** SD + Sil + R group showing astrocytes with GFAP immunoreactivity comparable to that of the control group. **F** Bar chart showing the mean ± SD of the GFAP immunoreactive area percentage per field in the different studied groups. Data are represented as mean ± SD (*n* = 8), group comparisons were performed using one-way ANOVA, followed by Tukey’s multiple-comparisons test. *P < 0.05, **P < 0.01, and ***P < 0.001 versus the control group; $*P* < 0.05, $$*P* < 0.01, and $$$*P* < 0.001 versus the SD group; #*P* < 0.05, ##*P* < 0.01, and ###*P* < 0.001 versus the SD + R group. SD: sleep-deprived untreated group; SD + R: sleep-deprived untreated group with recovery; SD + Sil: sleep-deprived group treated with silibinin; SD + Sil + R: sleep-deprived group treated with silibinin followed by recovery; GFAP: glial fibrillary acidic protein

GFAP-immunostained astrocytes of the control group exhibited a star-shaped morphology with thin processes. In contrast, astrocytes in the SD group appeared enlarged and had thick processes. Morphometric analysis revealed a significant increase in the GFAP immunoreactive area percentage in the SD group compared with the control group (*P* < 0.01). The SD + R group showed partial attenuation of astrocytic activation, with a significant reduction in GFAP immunoreactive area percentage compared with the SD group (*P* < 0.05). Similarly, silibinin treatment produced a more pronounced reduction in GFAP immunoreactivity, as demonstrated by the significant decrease in the SD + Sil group compared with the SD group (*P* < 0.001). The SD + Sil + R group exhibited astrocytic morphology approaching that of the control group, accompanied by a marked reduction in GFAP immunoreactive area percentage compared with both the SD group and SD + R group (*P *< 0.001). Moreover, no significant difference was observed between the SD + Sil + R and control groups (*P *> 0.05) (Fig. 7).

#### PCNA Immunostaining

**Fig. 8:**
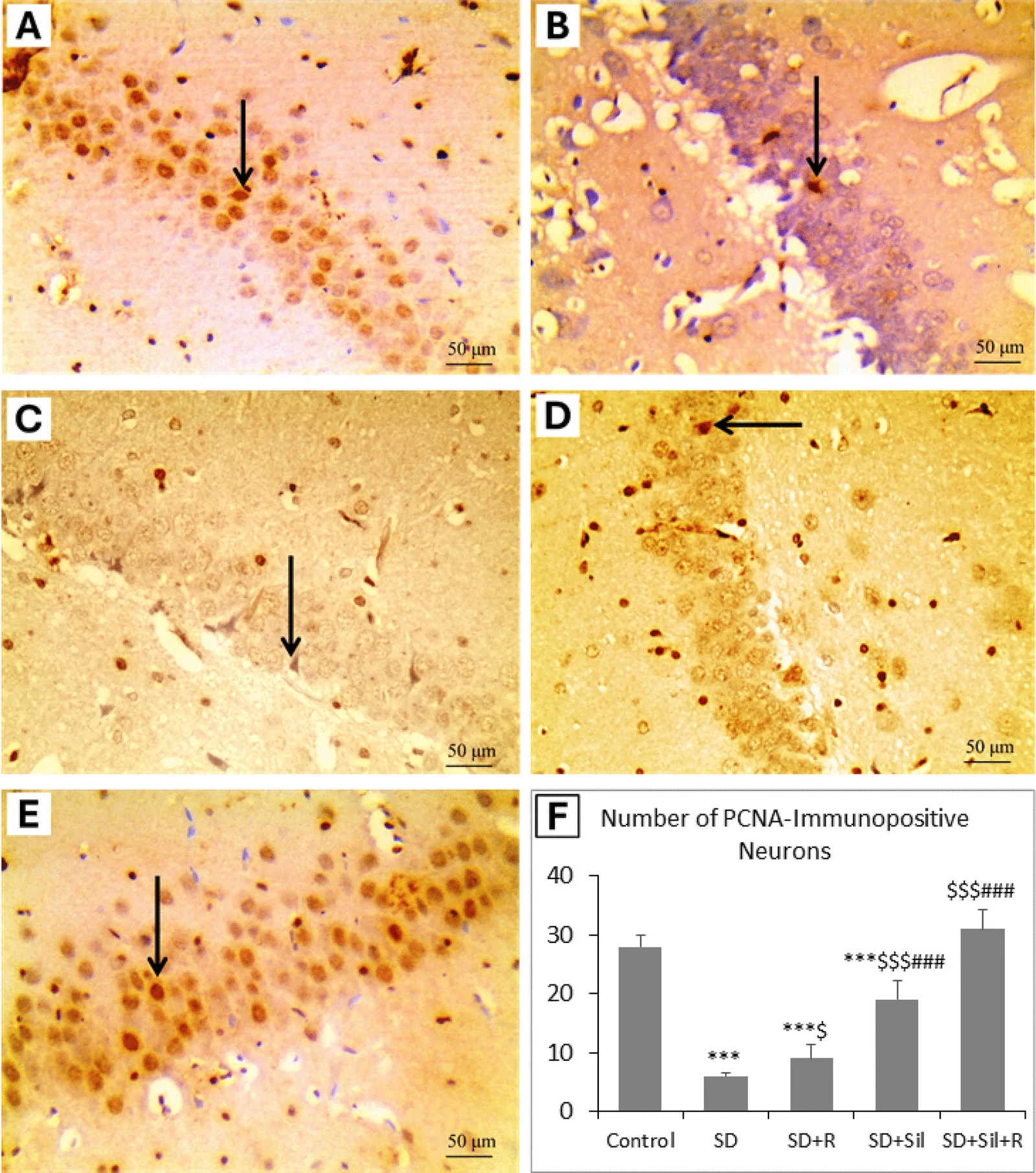
Representative photomicrographs of PCNA immunostaining of the CA1 region of the hippocampus (× 200; scale bar = 50 µm). **A** Control group showing intense positive PCNA immunoreactivity in numerous pyramidal neurons with dark brown nuclear reaction (arrow). **B** SD group showing PCNA immunoreactivity in a very few neurons (arrow). **C** SD + R group showing PCNA immunoreactivity restricted to some neurons (arrow). **D** SD + Sil group showing PCNA immunoreactivity in some neurons (arrows) in. **E** SD + Sil + R showing intense PCNA immunoreactivity comparable to that of the control group [F] Bar chart showing the mean ± SD number of PCNA-positive immunostained neurons per field in the different studied groups (*n* = 8). Statistical analysis was performed using one-way ANOVA followed by Tukey’s multiple-comparisons test. ***P < 0.001 versus control; $*P* < 0.05, $$$*P* < 0.001 versus the SD group; ###*P* < 0.001 versus the SD + R group. SD: sleep-deprived untreated group; SD + R: sleep-deprived untreated group with recovery; SD + Sil: sleep-deprived group treated with silibinin; SD + Sil + R: sleep-deprived group treated with silibinin followed by recovery; PCNA: proliferating cell nuclear antigen

The control group showed intense positive PCNA immunoreactivity, appearing as a dark brown nuclear reaction in several pyramidal neurons. In contrast, the SD group demonstrated a highly significant reduction in the number of PCNA-positive neurons compared with the control group. Quantitative analysis demonstrated a highly significant decrease in the number of PCNA-immunoreactive neurons compared with the control group (*P* < 0.001). Following recovery, the SD + R group showed partial restoration of PCNA immunoreactivity, with a significant increase in PCNA-positive neurons compared with the SD group (*P* < 0.05), although values remained significantly lower than the control group (*P* < 0.001). Silibinin administration resulted in a more pronounced improvement, as demonstrated by a significant increase in PCNA-immunoreactive neurons in the SD + Sil group compared with both the SD group and SD + R group (*P *< 0.001). The SD + Sil + R group showed the greatest restoration of PCNA immunoreactivity, with the number of PCNA-positive neurons significantly exceeding both SD and SD + R groups (*P* < 0.001). Furthermore, no significant difference was observed between the SD + Sil + R and control groups (*P* > 0.05) (Fig. 8).

#### Synaptophysin Immunostaining

**Fig. 9:**
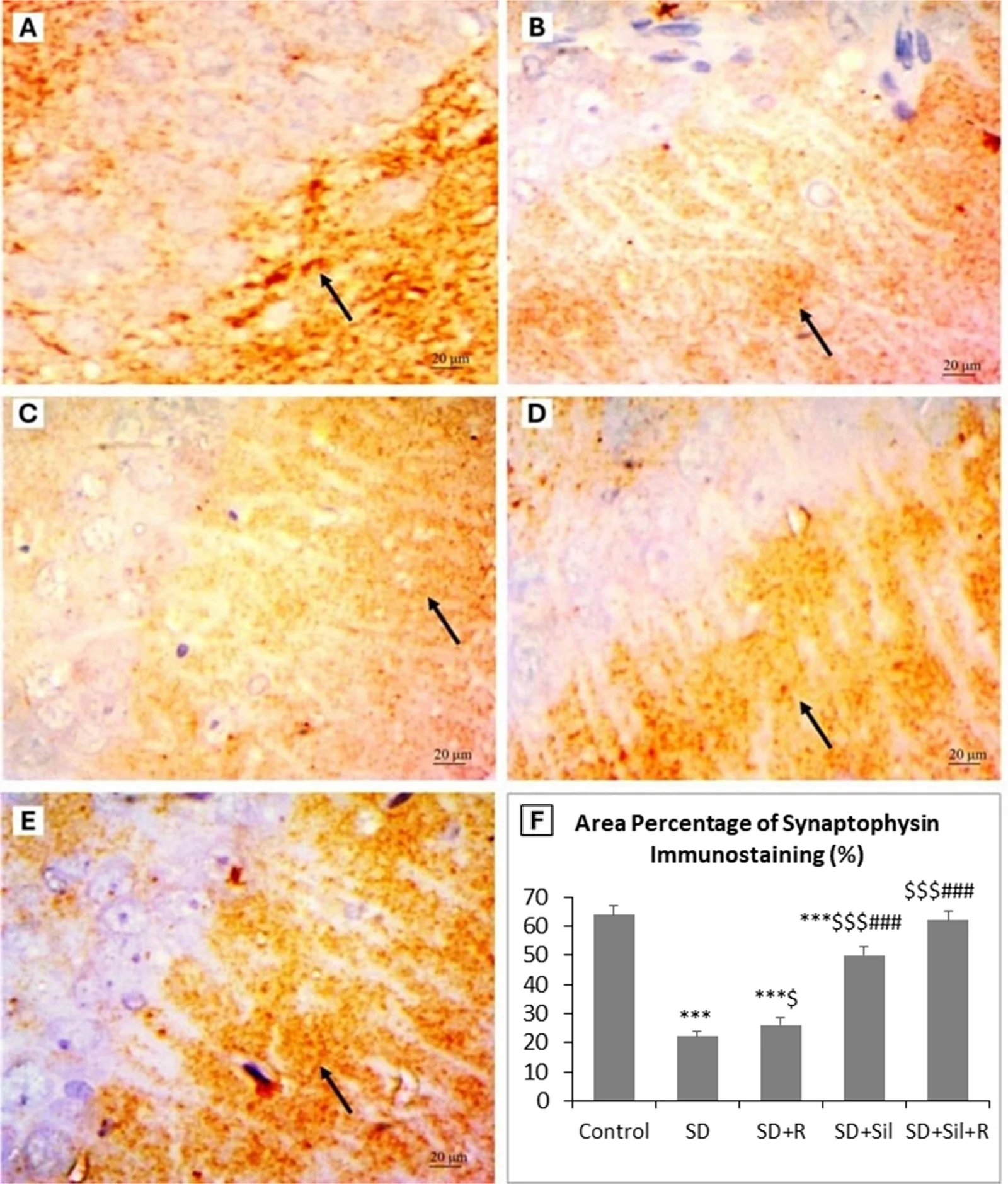
Synaptophysin-immunostained sections of the CA1 region of the hippocampus (Synaptophysin immunostaining, × 400; scale bar = 20 µm). **A** Control group showing intense synaptophysin-positive immunoreactivity as brown beaded staining surrounding the pyramidal neurons and within the neuropil (arrow). **B** SD group showing weak synaptophysin immunoreactivity with sparse brown beaded staining in the neuropil (arrow). **C** SD + R group showing weak synaptophysin-positive immunoreactivity (arrow). **D** SD + Sil group showing moderate synaptophysin-positive immunoreactivity (arrow). **E** SD + Sil + R group showing intense synaptophysin immunoreactivity surrounding the pyramidal neurons and within the neuropil, comparable to that of the control group (arrow). [F] Bar chart showing the mean ± SD of the synaptophysin immunoreactive area percentage per field in the different studied groups. Data are represented as mean ± SD (*n* = 8), group comparisons were performed using one-way ANOVA, followed by Tukey’s multiple-comparisons test. ***P < 0.001 versus the control group; $*P* < 0.05, and $$$*P* < 0.001 versus the SD group; ###*P* < 0.001 versus the SD + R group. SD: sleep-deprived untreated group; SD + R: sleep-deprived untreated group with recovery; SD + Sil: sleep-deprived group treated with silibinin; SD + Sil + R: sleep-deprived group treated with silibinin followed by recovery

Hippocampal sections from the control group showed intense synaptophysin-positive immunoreactivity, appearing as a brown beaded reaction on the surface of pyramidal neurons and in the neural synapses. The SD group displayed weak synaptophysin immunoreactivity with a significant reduction in synaptophysin immunoreactive area percentage compared with the control group (*P* < 0.001). The SD + R group showed moderate restoration of synaptophysin immunoreactivity, whereas the SD + Sil group exhibited a more pronounced increase. Morphometric analysis demonstrated a significant increase in the synaptophysin immunoreactive area percentage in SD + R and SD + Sil groups compared with the SD group (*P* < 0.05 and *P* < 0.001, respectively); however, their values were still lower than those of the control group (*P* < 0.001). Morphometric analysis revealed a significant increase in the synaptophysin immunoreactive area percentage in the SD + Sil + R group compared with the SD and SD + R groups (*P *< 0.001), with values comparable to those of the control group (*P* > 0.05) (Fig. 9).

## Discussion

Sleep deprivation is a significant physiological stressor that impairs cognitive function, particularly memory consolidation, and contributes to emotional disturbances such as anxiety-like behavior [45]. Although recovery sleep can partially restore cognitive performance and reduce hippocampal oxidative damage through restoration of cellular homeostasis [46], the extent of recovery after chronic sleep restriction remains incomplete. Silibinin has demonstrated neuroprotective properties in several experimental models through its antioxidant, anti-inflammatory, and cognitive-enhancing effects [47]. Therefore, the present study investigated the potential modulatory effects of silibinin on behavioral, histological, biochemical, and immunohistochemical alterations induced by 5 days of SD and a subsequent 24-h recovery in rats.

The present study demonstrated that chronic SD induced marked alterations in hippocampal function, including impaired cognitive performance, changes in oxidative and inflammatory markers, dysregulation of stress-related hormones, reduced neuroplasticity, and structural changes. A 24-h recovery period partially attenuated these alterations; however, silibinin treatment produced greater improvements, particularly when combined with recovery, resulting in near-complete restoration of several evaluated parameters. These findings suggest that silibinin attenuates SD-induced cognitive dysfunction and hippocampal injury and enhances recovery, which was associated with reduced oxidative stress and neuroinflammation, improved hormonal balance, and preservation of neuroplasticity.

In the present work, chronic SD was induced using the MMP method, a classic and widely used technique to induce animal models of paradoxical SD and impair memory in rodents [48]. Lack of sleep significantly impaired spatial memory and cognitive performance, as evidenced by the increased latency to first reach the former platform location during the probe trial of the MWM test and the reduced percentage of spontaneous alternation in the Y-Maze test. SD rats also exhibited anxiety-like behavior, reflected by reduced time spent in the center of the arena together with increased rearing, grooming, and defecation frequencies in the Open Field Test. These observations agree with previous studies demonstrating that SD disrupts hippocampal function, impairs spatial learning and memory, and enhances anxiety-like behavior [49–52]. These findings support the suitability of the MMP model for investigating the neuroprotective effects of potential therapeutic interventions, including silibinin.

The present study employed a 24-h recovery period to mimic the common pattern of weekday sleep restriction followed by weekend recovery. This recovery period partially improved cognitive performance and attenuated anxiety-like behavior; however, the SD + R group did not achieve complete normalization in the Morris Water Maze, Y-Maze, or Open Field Test parameters. This observation is consistent with previous reports indicating that a single weekend of recovery sleep is insufficient to fully reverse the cognitive and behavioral deficits induced by chronic SD [53]. Another study reported that after accumulating about 35 h of sleep loss over 5 days of restriction, animals recovered almost none of it, even after three full days of recovery [17]. Despite its clinical relevance, extending the recovery period may lead to a more complete restoration and could further enhance the neuroprotective effects of silibinin. Future studies should therefore investigate the impact of extended recovery durations, alone or in combination with silibinin, to determine whether longer recovery further enhances behavioral, biochemical, and histopathological outcomes. Notably, silibinin treatment significantly ameliorated the behavioral deficits induced by SD, improving spatial memory, spontaneous alternation, and Open Field Test performance. The combination of silibinin treatment and recovery produced the greatest behavioral improvement, with most parameters approaching control values. These findings are in agreement with previous studies demonstrating the neuroprotective effects of silibinin in experimental models of Alzheimer’s disease, cerebral ischemia, and formaldehyde-induced cognitive impairment [11, 14, 54].

Histopathological examination demonstrated that chronic SD induced marked structural alterations in the hippocampus, including a significant reduction in the thickness of the CA1 pyramidal layer, pronounced hypocellularity of the DG, and disorganization of the CA1 region. Similar findings have been reported by previous studies demonstrating that prolonged SD impairs hippocampal integrity and reduces neuronal proliferation within the DG [55]. Guzman-Marin et al. [56] reported that 96 h of SD in adult rats decreased cell proliferation in the DG and reduced the number of newly generated cells that matured into neurons, supporting the histopathological changes observed in the present study.

Consistent with the histopathological findings, a significant reduction in the number of PCNA-positive neurons and synaptophysin immunoreactivity in the hippocampus of SD rats was observed, indicating impaired hippocampal neurogenesis, reduced cell proliferation and/or DNA repair, and diminished synaptic density and plasticity. These alterations are consistent with the observed cognitive deficits and support the detrimental effects of chronic SD on hippocampal function [38]. Although a 24-h recovery period partially restored PCNA expression and synaptophysin immunoreactivity, complete normalization was not achieved. This is in line with those of Thome et al. [39], who reported that stress reduces synaptophysin expression, contributing to impaired synaptic plasticity and associated behavioral and cognitive deficits.

Our findings showed marked astrocyte activation in the hippocampus of SD rats, as evidenced by astrocytic hypertrophy and a significant increase in GFAP immunoreactivity. These findings indicate reactive astrogliosis and are consistent with previous reports showing that chronic SD induces astrocyte activation and morphological alterations, leading to impaired synaptic plasticity, release of proinflammatory cytokines, disrupted long-term potentiation, hippocampal dysfunction, and cognitive impairment [57–60]. Consistent with our findings, previous studies have also reported astrocytic hypertrophy and enhanced GFAP immunoreactivity following 5 days of SD, confirming that astrocytes are important targets of stress, with both chronic and acute stressors that can modify their morphology or affect the expression of various astrocyte-specific proteins, and undergo reactive astrogliosis in response to SD [61].

Silibinin treatment significantly improved the histopathological alterations induced by SD, as evidenced by reduced GFAP immunoreactivity, enhanced synaptophysin expression, and an increased number of PCNA-positive neurons. These improvements were most pronounced when silibinin was combined with a 24-h recovery period, with findings approaching those of the control group. Similar neuroprotective effects of silibinin have been reported previously and have been attributed to suppression of astrocyte activation, enhancement of synaptic protein expression, promotion of neuronal survival, reduction of neuronal apoptosis, and preservation of synaptic function through activation of the CaMKIIα signaling pathway [62, 63].

Biochemical analysis revealed that chronic SD not only negatively affects cognitive abilities in animal models but also triggers systemic neuroinflammation, oxidative stress, and lipid peroxidation, as evidenced by increased MDA, TNF-α, and IL-6 levels together with reduced SOD activity. Multiple studies have associated SD-induced memory impairment with hippocampal oxidative stress and neuroinflammation, which contribute to cognitive impairment and reduced neurogenesis within the DG [6, 64–66]. The 24-h recovery period partially restored these parameters but failed to normalize them, supporting previous reports showing that short-term recovery sleep only partially reverses the oxidative damage induced by SD [66, 67]. A study by Chen et al. [66] demonstrated that a single night of recovery only partially ameliorates oxidative stress, while even 1 week of recovery may be insufficient for complete restoration [67]. Normal sleep has been proposed to restore redox homeostasis by facilitating the removal ROS generated during wakefulness [68]. Accordingly, the incomplete recovery observed in the present study may reflect insufficient clearance of accumulated oxidative stress after only 24 h of recovery.

Oxidative stress and neuroinflammation are closely interconnected processes that amplify each other during SD. Excessive ROS promote lipid peroxidation and activate inflammatory pathways, leading to increased production of pro-inflammatory cytokines, including TNF-α and IL-6, which further exacerbate neuronal dysfunction, impair synaptic plasticity, inhibit neurogenesis, and contribute to cognitive decline [68–71]. These mechanisms are consistent with the behavioral deficits and hippocampal structural alterations observed in the present study. The hippocampus is especially vulnerable to oxidative stress owing to its intense metabolic activity and neuroinflammatory responses triggered by oxidative stress-activated microglia. Oxidative stress contributes to neuronal dysfunction through stress granule formation, sequestering essential proteins and transcripts, DNA damage, protein aggregation, and microglial activation that increases production of inflammatory cytokines, synaptic dysfunction, tissue damage, neurodegeneration, and inhibition of neurogenesis that play a significant role in cognitive impairment [69, 70].

Notably, silibinin treatment significantly attenuated oxidative stress and inflammatory responses, with the greatest improvement observed when combined with a 24-h recovery period. These findings suggest that the neuroprotective effects of silibinin are mediated, at least in part, through restoration of the oxidant-antioxidant balance and suppression of inflammatory signaling, thereby preserving hippocampal integrity and cognitive function. These findings are supported by previous studies demonstrating that silibinin possesses potent antioxidant, anti-inflammatory, and neuroprotective properties through modulation of multiple molecular pathways [9, 11, 72–74]. Accordingly, Tie et al. [75] proposed silibinin as a promising therapeutic candidate for neurodegenerative disorders.

The observed increase in proinflammatory cytokines may also contribute to dysregulation of the HPA axis. Previous studies have shown that proinflammatory cytokines can cross the blood–brain barrier, interfere with glucocorticoid receptor signaling, and disrupt HPA axis regulation, thereby perpetuating neuroinflammation and oxidative stress [71]. This bidirectional interaction between inflammation, oxidative stress, and HPA axis dysfunction may explain the hormonal alterations observed in the present study.

The present study demonstrated significant alterations in stress-related hormones following SD, including increased ACTH, corticosterone, and adrenaline levels, together with changes in growth hormone and insulin levels. These findings indicate activation of the stress response system and disruption of endocrine homeostasis following chronic SD. Consistent with our results, previous studies have reported increased norepinephrine, ACTH, and corticosterone levels after 24-h of SD, with partial or complete restoration following 24-h of recovery sleep [76, 77].

Excessive or prolonged activation of the HPA axis and adrenergic system can impair hippocampal function by disrupting glucocorticoid feedback regulation, reducing hippocampal resilience, and impairing memory-related processes. Sustained overproduction of glucocorticoids diminishes the HPA axis's response over time, leading to disruption of hippocampal negative feedback mechanisms and resistance to glucocorticoids [78, 79]. Stress-induced glucocorticoid elevation has been shown to impair hippocampal-dependent memory and cognitive performance [80, 81], while a direct relation between cortisol levels and hippocampal size in connection with mood disorders has been demonstrated. Furthermore, blocking the elevation in glucocorticoids in animals deprived of sleep for 72 h entirely prevented the reduction in cell proliferation and neurogenesis, highlighting the pivotal role of stress hormones in neuronal injury [82].

The observed hormonal dysregulation may also explain the reduction in neuroplasticity-related markers observed in the present study. Elevated glucocorticoid levels have been associated with decreased BDNF expression, resulting in impaired synaptic plasticity, neurogenesis, and cognitive function [83, 84]. Likewise, previous studies have shown that 8 h of SD reduces CaMKII gene expression, thereby impairing synaptic plasticity, short-term memory, and long-term potentiation [85]. Therefore, the reduced BDNF, synaptophysin, and CaMKII levels observed in our study may represent downstream consequences of HPA axis activation and contribute to the spatial memory deficits induced by chronic SD.

The present study demonstrated a significant reduction in serum growth hormone levels following chronic SD. Although a 24-h recovery period produced a slight increase in growth hormone levels, this change did not reach statistical significance. Silibinin treatment significantly improved growth hormone levels, with further enhancement when combined with recovery. Growth hormone is a critical mediator linking sleep regulation with the maintenance of normal hippocampal synaptic function and is essential for the maturation and presynaptic strengthening of engram cells required for memory formation and recall [83]. The observed reduction in growth hormone levels is consistent with previous reports showing that SD disrupts the normal nocturnal growth hormone surge [84]. Excessive glucocorticoid secretion, together with the increased oxidative stress and inflammatory responses observed in the present study, may contribute to suppression of growth hormone release by reducing hypothalamic growth hormone-releasing hormone (GHRH) expression, decreasing pituitary responsiveness to GHRH, and enhancing somatostatin secretion [85, 86]. The partial restoration of growth hormone levels following recovery and, more prominently, after silibinin treatment may therefore have contributed to the improvement in hippocampal function and cognitive performance observed in the present study.

Insulin signaling is also important for neuronal development, feeding behavior, and cognitive processes. Insulin exerts direct effects within the hippocampus to facilitate memory processes through activation of the phosphoinositide 3-kinase (PI3K) signaling pathway, whereas inhibition of PI3K impairs memory function [86]. In the present study, chronic SD increased serum insulin levels, while recovery and silibinin treatment partially restored them toward normal values. Cortisol, oxidative stress, and inflammation have been shown to impair insulin signaling at early time points, thereby contributing to deficits in learning and memory [87]. Consistent with our findings, Buxton et al. [88] reported that short sleep duration significantly reduces insulin sensitivity and impairs glucose tolerance. Therefore, the elevated insulin concentrations observed in the SD rats in our study may reflect a compensatory response to reduced insulin sensitivity induced by chronic SD [89].

A 24-h recovery period was insufficient to fully restore ACTH, corticosterone, adrenaline, growth hormone, and insulin levels to baseline values, consistent with previous reports demonstrating persistent endocrine dysregulation following short-term recovery from SD [90–93]. In contrast, silibinin treatment significantly attenuated the SD-induced alterations in stress-related hormones by reducing ACTH, corticosterone, adrenaline, and insulin levels while increasing growth hormone levels. Previous studies have likewise demonstrated that silibinin improves insulin sensitivity, attenuates diabetic complications, and protects against organ damage [94].

The present study is subject to certain limitations that should be considered when interpreting the findings. First, a silibinin-only control group was not included; although previous studies have generally reported that silibinin exerts minimal physiological and cognitive effects in healthy rodents at comparable doses, the inclusion of a silibinin-only group would have allowed direct confirmation that the observed effects resulted specifically from attenuation of SD-induced alterations rather than from any intrinsic pharmacological actions of silibinin under normal physiological conditions. Future studies should include such a group to strengthen the interpretation of the findings. Second, although the selected silibinin dose (100 mg/kg) was supported by previous studies demonstrating neuroprotective effects, a dose–response study was not performed in the current sleep deprivation model. Future studies are needed to determine the optimal dose and administration regimen of silibinin for this condition. Third, sleep architecture was not monitored using electroencephalography; therefore, the degree and specific stages of sleep disruption could not be objectively verified. Fourth, the Morris water maze assessment was limited to the latency first to reach the former platform location during the probe trial, while additional behavioral parameters, including time spent in the target quadrant, swimming speed, and escape latency during the acquisition phase, were not assessed.

In conclusion, chronic SD induced cognitive impairment associated with oxidative stress, neuroinflammation, hormonal dysregulation, impaired neuroplasticity, and structural alterations in the hippocampus. A 24-h recovery period partially improved these alterations but did not fully restore them. Silibinin administration significantly attenuated the behavioral, biochemical, histopathological, and immunohistochemical changes induced by SD by reducing oxidative stress, inflammation, and stress hormone levels while restoring the expression of BDNF, synaptophysin, and CaMKII and preserving hippocampal structure and synaptic plasticity. The combination of silibinin with a 24-h recovery period produced additional improvement, with several parameters approaching control values. These findings suggest that silibinin may exert neuroprotective effects against SD-induced hippocampal injury and may have potential as an adjunctive approach to enhance recovery from sleep loss; however, further studies are required to evaluate its optimal dosing, long-term safety, and translational relevance.

## Data Availability

Data are available from the corresponding author upon request.
